# “Prideful Apathy”: A Phenomenological-Psychopathological Study of Emotion Engagement and Regulation Tasks

**DOI:** 10.3390/brainsci16010080

**Published:** 2026-01-07

**Authors:** Aleš Oblak, Sara Rigler, Liam Korošec Hudnik, Jurij Bon

**Affiliations:** 1 Laboratory for Cognitive Neuroscience and Psychopathology, Center for Clinical Psychiatry, University Psychiatric Clinic Ljubljana, 1260 Ljubljana, Slovenia; 2 Center for Cognitive Science, Faculty of Education, University of Ljubljana, 1000 Ljubljana, Slovenia; 3 Department of Psychiatry, Faculty of Medicine, University of Ljubljana, 1000 Ljubljana, Slovenia; 4 Department of Psychology, Faculty of Arts, University of Ljubljana, 1000 Ljubljana, Slovenia

**Keywords:** emotion regulation, emotion processing, phenomenology, depression, anxiety, ecological validity, magical thinking

## Abstract

**Background/Objectives**: Emotion dysregulation is central to many psychiatric disorders. Laboratory-based tasks designed to assess emotion processing and regulation often rely on standardized affective stimuli whose ecological validity remains unclear. We contextualize this study in our broader research program of neurophenomenological reflection of standard paradigms in experimental cognitive psychology. **Methods**: This study investigates the lived experience of 27 patients with affective disorders as they performed a cognitive-affective task combining working memory demands with exposure to negative emotional images. Phenomenological interviews were used to collect data on their experience of the task. **Results**: We identified three key experiential domains: whether the stimuli are capable of eliciting a spontaneous emotional response, voluntary construction of an emotional responses, and its temporal dynamics. Patients reported on two alterations in affectivity that are associated with dysregulation: (a) affective enchantment, characterized by intense emotions combined with superstitious appraisal; and (b) disintwinement (a sense of detachment and emotional blunting). Emotional responses exhibited complex unfolding across moment-to-hour timescales, sometimes persisting and blending across trials (impressionability), reflecting clinical phenomena such as rumination. Additionally, patients employed a range of explicit and implicit regulation strategies, many acquired through therapy or long-term coping. **Conclusions**: Our findings reveal the limitations of rapid, static image-based paradigms in eliciting authentic and spontaneous affectivity in clinical populations, highlighting the need for more ecologically valid experimental designs. Furthermore, inclusion of reports on such subtle affective states as vital feelings in laboratory-based experimental assessments is necessary for a comprehensive understanding of altered phenomenology of affectivity in affective disorders.

## 1. Introduction

Emotion regulation encompasses the cognitive and social processes by which we influence the occurrence, duration, and magnitude of an emotional response [1,2]. The most well-developed model of emotion regulation is the process model put forward by Gross [3]. It consists of five components that are dependent on the temporal dynamics in relation to the emotional stimulus: (i) situation selection; (ii) situation modification; (iii) attention deployment; (iv) cognitive change; and (v) response modulation. Originally considered to reflect a linear development of determinate events, the process model has since been extended to include the non-linear, cyclical interplay between these five stages [4].

Several taxonomies of emotion regulation strategies have been proposed [5,6]. Emotion regulation strategies can be antecedent (e.g., selecting ahead of time the least emotionally salient stimuli) or response-focused [7]. They can be active or passive [8]. If they support mental wellbeing or are detrimental to it, they can be adaptive or maladaptive, respectively [9,10]. Finally, depending on whether they pertain to the regulation of incoming bodily feelings (e.g., through progressive muscle relaxation) or manipulation of cognitive appraisals, they can be bottom-up or top-down, respectively [11]. Evidence indicates that existing classifications of emotion regulation strategies often group them together despite being supported by distinct neurocognitive mechanisms. For example, brain imaging studies reveal different activation patterns for detachment versus reappraisal strategies [12], and meta-analyses suggest a wide heterogeneity within key regulatory types [13]. This overlap leads to ambiguous conclusions and challenges in comparing findings across studies, underscoring the urgent need for a more precise taxonomy of emotion regulation strategies. The present study aims to shed light on this issue by presenting the lived experience of a laboratory-based emotion processing and regulation task in patients with affective disorders.

Emotion dysregulation is a symptom that cuts across psychiatric diagnostic categories [14,15,16,17]. It is associated with poor treatment outcomes and heightened risk of suicidality [18]. Despite extensive research into the mechanisms of emotion dysregulation, management of this symptom remains poor [19]. Emotion dysregulation forms both a core and a peripheral symptom in several psychiatric disorders [20]. Depression, for example, is characterized by both the inability to upregulate dysphoria, hypo- and anhedonia [21], and patterns of affective down-regulation such as ruminating [22,23,24], which prolong depressive episodes and are associated with reduced likelihood of remission across treatment modalities [25,26]. Anxiety is characterized by the inability to regulate away fear, as well as preponderance for upregulating it through worrying [27,28]. Emotional dysregulation is the core symptom of various personality disorders, such as borderline personality disorder [29], where emotions are characterized by their all-encompassing phenomenology, diminished sense of self [30], and an incontrollable excess of energy that has been termed desperate vitality [31].

In affective disorders, patients often rely on maladaptive strategies such as rumination and denial, since complex cognitive strategies are not necessarily accessible to them. This is true both on the level of specific strategies that people use to regulate their emotions (e.g., cognitive reappraisal is broadly considered to be adaptive, whereas affective suppression is a maladaptive emotion regulation strategy) [32,33], as well as how they conceptualize and experience their affectivity [34], as is the case in alexithymia. People higher in alexithymia are more likely to engage in maladaptive emotional regulation strategies. More specifically, when we fail to recognize our own emotions, we are more likely to engage in avoidant strategies (e.g., expressive suppression, withdrawal, and denial) and less likely to employ adaptive strategies such as cognitive reappraisal, active problem-solving, and seeking social support [35].

In experimental cognitive psychology, emotion processing is typically measured by exposing participants to a series of affective stimuli. Commonly, they consist of static images, derived from standardized libraries of such stimuli [36,37,38]. Performance measures are usually based on the circumplex model of emotion processing [39] and thus consist of valence and arousal. While emotionally salient movies are also commonly used as stimuli [40,41,42,43], static images are favored, especially in studies relying on neuroscientific methods (e.g., electroencephalography and functional magnetic resonance imaging), as many techniques of analyzing neural signals require precisely time-locked experimental events [44]. Despite their limitations, time-locked signals have proven useful in identifying neural correlates of emotion processing [45,46,47]. However, a major problem with such studies is that genuine emotional processing as well as imagining emotional situations are associated with the same patterns of neural activation [48,49], and there has been poor replication of some of the observed effects [50,51].

In recent years, phenomenological methods have been introduced into cognitive neuroscience and experimental cognitive psychology [52,53,54,55]. Integrating qualitative phenomenological insights and neuroscientific studies has revealed that we can often see one-to-many mapping between psychological tasks and associated experiences (e.g., how the same task can be performed using multiple cognitive strategies) [56,57,58,59]; unexpected experiences elicited by the tasks (e.g., intense emotions while performing a working memory task) [60], and certain paradigms (e.g., thinking-on-demand) lacking ecological validity [61]. Integrating phenomenology and experimental cognitive psychology has been extended into clinical settings [62,63,64]. Interview-based qualitative phenomenology has thus emerged as an important source of data, reflecting on the validity of laboratory-based tasks, as well as additional information aiding in the interpretation of experimental findings [65].

### 1.1. Reflexivity Statement: Towards a Cognitive Anthropology of Experimental Cognitive Psychology

By way of reflexivity statement, we will briefly outline our research program that we have been developing over the last decade. *Neurophenomenology* is the methodological proposal put forward by Varela [55] for solving the so-called *hard problem of consciousness* [66]; that is, the question of how conscious experience can arise from non-conscious matter. As a solution, Varela [55] suggests systematically integrating phenomenology (i.e., the philosophical study of consciousness) and neuroscience; or, phrased in the jargon of neurophenomenology, mutually constraining first- and third-person data [67].

Over the years, several approaches in neurophenomenology have emerged. Some researchers practice it as a purely theoretical framework [68,69,70]. Others focus on collecting first- and third-person data simultaneously [56,71]. The first neurophenomenological study was conducted by Lutz et al. [56], where they demonstrated that different subjectively reported strategies on how a simple optical illusion is manipulated are associated with different electroencephalographic signatures. Over the years, a large body of different research designs has been developed under the heading of neurophenomenology. One of them, which we take as our starting point, is so-called *front-loaded phenomenology.* Front-loaded phenomenology consists of first obtaining detailed descriptions of a given aspect of experience (either through philosophical reflection or qualitative research) and then operationalizing them in a controlled psychological or neuroscientific experiment [54].

Ten years ago, our group started using this approach to systematically investigate how standard paradigms in experimental cognitive psychology are experienced by our participants. We started by investigating the performance of simple arithmetic tasks [72]. This was followed by an in-depth investigation of the lived experience of working memory tasks, where we were particularly interested in the change detection task and the visual span task. In separate studies, we investigated the lived experience associated with these paradigms [57,58], how the phenomenological differences are reflected in participants’ behavioral performance [59], how the experience operationalized by a standardized cognitive task differs from when working memory is deployed in a naturalistic setting [73], and finally how these phenomenological differences (primarily operationalized as different cognitive strategies) recruit different neural mechanisms [74,75]. In addition to conducting empirical studies, we periodically published technical papers for integrating phenomenology and cognitive science based on emergent methodological insights [76,77]. This multifaceted approach allowed us to posit an integrated view of working memory [78]. Our group extended this work to false memory paradigms [79]. In short, we are endeavoring a *cognitive anthropology of experimental cognitive psychology.*

Through this work, we gradually developed a methodology that attempts to accommodate the epistemological constraints of “the empirical turn” in phenomenology [52,53,80]. We are primarily integrating the two most commonly employed phenomenological approaches in cognitive science: *descriptive experience sampling* (DES) and the *micro-phenomenological interview* (MPI). DES is based on disrupting a person’s flow experience and having them report on what was present in their consciousness immediately before the prompt, thereby minimizing the reconstructive effect of retrospection [81]. We are operationalizing this methodological move by having participants perform a block of a cognitive task, which terminates after a random trial. The interview is then centered on the final trial. Second, MPI is a qualitative technique that focuses on both how a person’s experience evolves in time (*diachronic dimension*) and a detailed description of how it is present in a given moment (*synchronic dimension*) [82,83,84]. MPI is geared towards describing lived experience at the time scale of a single action–perception cycle (i.e., an interval of around 300 ms) [85]. Such detailed descriptions make it amenable to theorize about cognitive (sub)processes that may play a role in a given aspect of experience.

When conducting phenomenological studies, we subscribe to a constructivist epistemology. Constructivist epistemology does not consider data collection to uncover a pre-given reality but as a result of a mutual negotiation of meaning between the participants and the researchers [86,87]. As such, we often present data that are highly fragmented, reflecting distinction between similar phenomena that are appraised as relevant by our participants, as well as employ neologisms where they feel standard scientific terminology does not do justice to their experience. Here, we are building on the classic works of philosophical phenomenology where novel terms were invented with the express purpose of distancing readers from how they ordinarily think of their experience and consider it in a new light [88,89,90].

All of the authors of this paper work in a psychiatric setting; as a researcher and TMS technician [AO], a clinical psychologist [SR], and psychiatrists [LKH, JB]. Thus, we started to integrate this approach into the study of psychopathology. We are operating within the paradigm of *interventional psychiatry.* It emphasizes early interventions into the trajectory of psychiatric disorders, preventing them from becoming chronic conditions. It is focused on identifying dimensional mechanisms of human functioning (e.g., emotion dysregulation) rather than focusing on specific diagnostic categories. It draws on how these potentially aberrant processes are implemented in specific neurocognitive networks [91]. Unlike traditional reactive approaches, interventional strategies aim to prevent symptom progression by modulating neural plasticity, offering hope for more precise, holistic care that bridges biological vulnerabilities with psychological and social factors.

Interventional psychiatry assumes explanatory pluralism; an epistemological stance that attempts to integrate several levels of description in understanding the occurrence and treatment of psychopathologies without treating any of them as epistemologically superordinate [92]. Taylor et al. [91] point out that current approaches in psychopathology tend to paint a fragmented account of psychiatric disorders (what they termed *neuro-cubism*), where different perspectives (biological, psychological, and social) co-exist (and sometimes overlap), but are rarely integrated into a unified whole. This problem is particularly salient when it comes to phenomenology, which is typically entirely omitted from integrative models of psychiatric disorders [93]. This is a shortcoming that we hope to address with our methodology. Since emotion processing tasks are commonly used in interventional psychiatry to (a) understand psychopathological processes; (b) assess outcome measures following treatment; and (c) personalize treatment, we sought to employ our methodology to reflect on such paradigms as well.

### 1.2. How It Feels to Perform a Cognitive-Affective Task

This paper aims to provide a detailed description of the lived experience of patients with affective disorders as they perform an emotion processing task. Following the American Psychological Association’s guidelines for reporting qualitative studies [94], we split the presentation of our findings into several texts. Elsewhere, we have already presented our findings from the normative population [95]. We investigated their lived experience using laboratory-based tasks that used standardized images to elicit emotions. We found that such images often fail to elicit genuine or spontaneous emotional reactions. Following LeDeux and Damasio [96], we termed this capacity of environmental stimuli affective competence.

Furthermore, in the context of a laboratory-based experimental cognitive psychological task, such stimuli are presented in rapid succession, each lasting for a handful of seconds. As such, our participants were commonly not sure what the images actually depicted (we termed this experience a gap in sensemaking). The lack of spontaneous emotional response and clear understanding of what is depicted in the image prompted our participants to disambiguate the stimuli. This was supported by the experiential process of sensemaking. We borrowed the term sensemaking from Sandberg and Tsoukas [97], who conceptualize it as an integrated experience, consisting of sensorimotor coupling with the world, emotional response, as well as establishing narrative-level conceptual understanding (see also emotion schema in psychotherapeutic literature) [98,99]. Sensemaking was related to visual examination of stimuli, searching for affectively charged details, while, at the same time, constructing a story about them. Importantly, participants’ emotional response was subjectively related to their self-constructed narrative, rather than to the stimuli themselves.

Replicating findings from earlier phenomenological studies of emotions [100,101], we found that emotional response is experienced as evolving in time, consisting of anticipation, inkling (an immediate emotional response, consisting of vague bodily feelings and characterized by valenced, but not yet namable, basic emotions), development (i.e., emergence of namable emotions), and finally termination. For many participants, the emotions persisted from one trial into the next. This emotional persistence was occasionally so strong that it led to what we termed impressionability, a long-lasting change in mood. Importantly, while many participants reported their emotional responses on a trial-by-trial basis as self-constructed, impressionability was experienced as spontaneous.

Finally, while we did observe aspects of experience that could be analytically framed as emotion regulation strategies, these amounted to weak qualitative categories. Many were idiosyncratic or were reported by only a handful of participants. Furthermore, categories such as distancing were established as a consequence of poor phenomenological aptitude (i.e., participants not being able to describe their experience in a more detailed way) [102] or were, through subsequent inquiry, revealed to relate more closely to other aspects of experience (e.g., low sensemaking).

## 2. Materials and Methods

### 2.1. Participants

Thirty patients (twenty women) signed an informed consent form to participate in this study. The participants were between 22 and 52 years old (mean = 34.6; *SD* = 11.6) and had completed on average 19.4 years of education (*SD* = 5.4). Three patients (all women) were excluded from the study because they had a history of psychosis. According to the criteria laid out in the International Classification of Diseases, tenth revision (ICD-10), twenty-two patients had a diagnosis of major depressive disorder and sixteen of generalized anxiety disorder. Four had comorbid borderline personality disorder and two had comorbid eating disorders. The diagnosis was made by their attending psychiatrist. All patients were receiving active treatment (psychotherapy, psychiatric medication, or neuromodulation therapy) at the time of the study. All the participants signed an informed consent to participate in the study. This study was approved by the Committee for Medical Ethics of Republic of Slovenia.

### 2.2. Ethical Considerations

Qualitative research of the kind presented in this paper includes additional ethical considerations. Patients signed additional forms consenting not only to their participation in the study but for audio recordings of the conversations to be made and direct anonymized quotes to be used in any publications. As will be shown below, patients came in for several interviews. At the beginning of each interview, we once again verbally asked them for their consent for continuous participation as well as for us to start recording our conversation. Since patients with affective disorders represent members of a vulnerable population, the interviews were conducted in-person at a psychiatric clinic. In case of adverse effects of participating in the study, the patients were offered continuous psychosocial support. It is to be noted that the patients found participation in the study intriguing, pointing out that they learned something new about themselves in the process.

### 2.3. Protocol

Participants completed a computerized cognitive-affective task that integrated emotion engagement or regulation with concurrent cognitive load via an embedded n-back working memory component. This dual-task design allowed targeted investigation of (a) interactions between executive functions and emotion processing; (b) potential attentional bias toward the ostensibly primary affective task; and (c) naturalistic scenarios where emotional reactions interweave with ongoing cognition. Each trial opened with a 2.0 s fixation cross followed by a 6 s presentation of a randomly selected affective image at screen center (for images used, consult Supplementary Materials).

Participants were instructed either to experience the emotions evoked by the image freely (emotion *engagement*) or to modulate their response to achieve affective neutrality (emotion *regulation*). In the final 2 s of image display, a letter overlay appeared, requiring an evaluation of whether it matched the letter from two trials earlier. Responses followed a 1 s blank interstimulus interval, after which participants rated their valence and arousal on separate 9-point Self-Assessment Manikin (SAM) scales [103] via left-click selection and right-click confirmation. A 2 s intertrial interval then advanced to the next trial.

Implemented in a custom PsychoPy (version 2025 1.1) script [104], the task supported a 2 × 2 factorial design crossing emotional task (engagement vs. regulation) and working memory load (*0-back* vs. *2-back*), with each condition run in a dedicated block. Valence, arousal, accuracy, and reaction time data were recorded but are not analyzed here, as this requires specific methodological considerations that extend beyond the scope of this paper [78].

**Figure 1:**
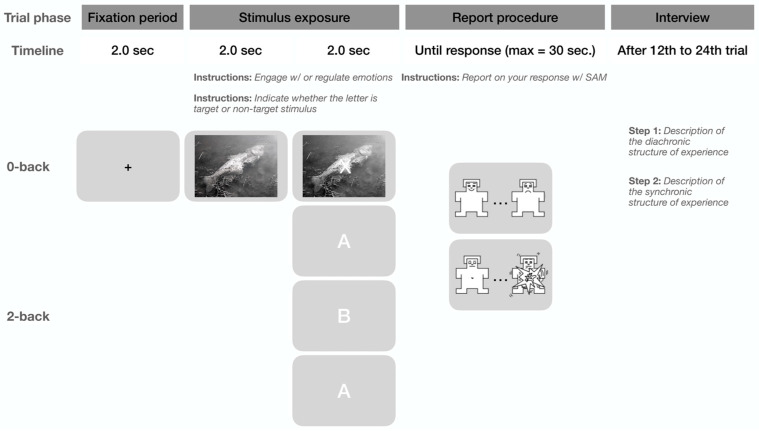
The structure of the cognitive-affective task.

Affective conditions varied by instruction: engagement encouraged uninhibited emotional responding, while regulation prompted neutralization without prescribing strategies (e.g., distancing or reappraisal). The *n-back* task overlaid white Helvetica letters on the image. In 0-back, right-button responses targeted ‘X’ and left-button non-targets; in *2-back*, right-button hits matched the letter from two trials prior, with left-button for mismatches (see Figure 1). Exact instructions given to the participants are made available in Supplementary Materials.

Participants completed four sessions across separate days (≤1 week apart), each devoted to one randomized condition in a single block of 12–24 trials. The task auto-terminated at a random trial endpoint within this range, informed by prior work [95] prioritizing experiential depth in phenomenological probes of experimental paradigms over high-volume sampling. This trial count, per earlier findings [57,58,78], facilitates task immersion, minimizing artifacts like initial awkwardness or anxiety. Post-task phenomenological interviews based on MPI were conducted by a researcher formally trained in this method. The interviews were recorded on an Olympus WS-852 (OM Digital Solutions, Tokyo, Japan) digital audio recorder.

### 2.4. Analysis

The analysis followed *constructivist grounded theory* [86]. Accordingly, we employed *parallel analysis:* data collection and analyses processes took place simultaneously, mutually informing each other [105]. As new insights emerged from the analysis, this allowed us to test them by modifying the questions in subsequent interviews (with both old participants who came in for follow-up interviews and new participants). Interview recordings were transcribed verbatim. No transcription software was used as the process of transcribing itself represents an analysis step, forcing the analyst to refamiliarize themselves with the qualitative material anew. The main analytical instrument that we employed was *coding:* the ascription of general, descriptive tags to sections of raw text based on their conceptual similarity. Coding was conducted in Delve, a qualitative analysis software. Three types of coding were used: inductive coding (wherein tags are assigned based purely on the raw data rather than resorting to pre-existing theories), inductive–deductive coding (where the taxonomy that had been established during inductive analysis is used to fit new qualitative material onto pre-existing codes, while keeping an open mind to where incoming data challenges our emergent understanding), and finally, deductive coding was used (the final taxonomy of codes was fitted to all of the qualitative material).

In the final stage of the analysis, an annotated codebook was constructed [106]. In qualitative research, a codebook is a document in which all the experiential categories constructed during the analysis are described. Each entry contains the following elements: (i) a telling name; (ii) a definition; (iii) relationship to other codes; (iv) relevant quotes; and (v) additional considerations. The codebook serves four purposes: (1) it makes the analysis process tractable; (2) it allows independent researchers to familiarize themselves with the analysis procedure; (3) it allows for the construction of new instruments (e.g., questionnaires); and (4) a logically well-ordered codebook represents a criterion of validity. The relevant interview quotes were translated by the principal investigator [AO], who is a cognitive scientist by training and has both studied and worked in the anglophone world. Thus, particular attention was paid to the faithfulness of the translations to the original meaning as well as subtle nuances in the language used. Furthermore, in this paper, only the most salient quotes are used.

The experiential categories yielded by coding were described in an annotated codebook, which also includes an exhaustive list of supporting quotes (available at https://osf.io/qsjua/).

In the codebook, we organized the experiential category into levels of coding. These refer to the degree of abstraction from the raw data, with the lowest level representing similarity to raw text (i.e., comes to closest to the wordings that our participants used) and the highest level of coding representing attempts at constructing more generalizable theoretical constructs. The number of levels of coding was determined empirically, based on the conceptual similarity between the experiential categories.

**Figure 2:**
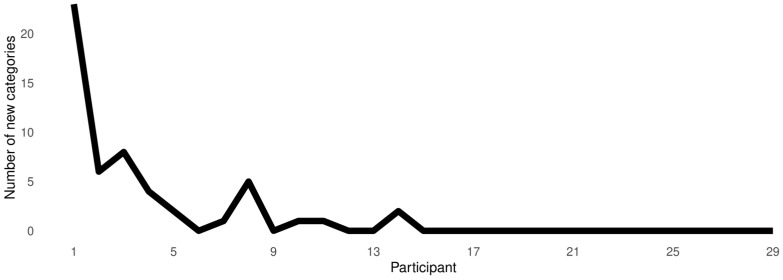
Saturation grid.

Three criteria of validity were used. First, *consensual validation* was employed. As participants came in for subsequent sessions, they were explicitly asked about our coding procedure. Categories were considered valid only if the participants agreed that they adequately represent their experience. Second, *intercoder verification* was used. Two researchers, both formally trained in several techniques in qualitative research [AO & SR] analyzed the data independently. Given the complexity of the qualitative material yielded by the techniques we used [52], a quantitative comparison was untenable. Thus, the analysts discussed the differences in their categories before agreeing to a taxonomy that accounted for the entirety of the qualitative material. Third, data were collected and analyzed via iterative coding until saturation was reached (see Figure 2). In qualitative research, saturation (also known by newer term *conceptual depth*) is the point at which a sufficient amount of data are collected to (a) warrant theory construction; and (b) rendering subsequent collection unnecessary [62,107]. We used a saturation grid to make this determination. A saturation grid is a tabulation where subsequently recruited participants are depicted on the *X*-axis and the number of newly observed categories is depicted on the *Y*-axis. When no new codes (i.e., when some observed phenomenon does not fit into an already-existing code, a new one has to be inductively constructed) are observed over several participants, saturation (i.e., the point were further recruitment of participants is unnecessary) is considered to be reached [62,107]. In our case, saturation was reached following the 14th participant. Nonetheless, we continued with recruitment so as to avoid a false stop.

## 3. Results

**Figure 3:**
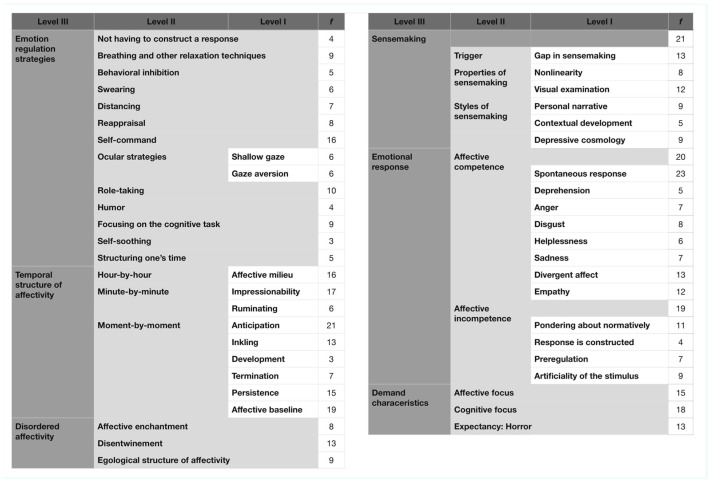
Taxonomy of the experiential categories. Experiential categories are presented in three coding levels, with level I representing the lowest and level III the highest degree of abstraction of raw data. Frequencies indicate the number of participants in which a particular experiential category was observed collapsed across interviews.

A total of 101 admissible interviews (on average 3.7 per participant) were conducted. The analysis of the qualitative material yielded 51 codes, which were subsequently organized into three levels of coding. These refer to the degree of abstraction from the raw data, with level 1 denoting the smallest and level 3 the highest degree of abstraction (in this text, experiential categories are written out in italics with the level of code added in the subscript). Six level 3 categories were observed: (i) *emotion regulation strategies*; (ii) *temporal structure of affectivity*, describing how the experience of a single trial of the cognitive-affective task and likely reflecting the methodological commitments of MPI; (iii) *disordered affectivity*, describing the phenomenological Gestalt of affective disorders; (iv) *sensemaking*, describing the experiential process whereby emotional responses were voluntarily constructed; (v) *emotional response*, describing participants’ experience of the stimuli; and (vi) *demand characteristics*; that is, how the constraints of the laboratory-based setting were reflected in their experience. The taxonomy of experiential categories is displayed in Figure 3. 

To orient the reader in the qualitative material, we will begin the presentation of our findings with a *composite description* (i.e., a speculative synthesis of a typical experience) of a patient’s experience of performing a cognitive-affective task [108]. This experience is characterized by what one patient described as *prideful apathy.* While she used this term to describe a specific emotion regulation strategy (see more on this below), we believe that it encapsulates the general experience of the cognitive-affective task as well. The stimuli derived from standardized libraries of affective pictures rarely proved to be capable of eliciting a genuine emotional response. This meant that there was but minimal difference in the experience of engagement and regulation conditions. Furthermore, since in the context of a psychological study, there has to be something to be measured, the participants felt compelled to willfully construct an emotional response. They did this by imagining a narrative surrounding the stimulus. Nevertheless, we observed two experiential dynamics that veered away from this description. These appeared not as specific moments of experience, but phenomenological Gestalten, typical way of being-in-the world. For some patients, the world appears as emotionally distant with every event seeming as if receding into the background. On the other hand, for some, emotionally salient events appeared to be hypertrophic in their mind. From a third-person researcher perspective, we could characterize this process as being related to a kind of superstition: some patients felt that merely by looking at the image (and later by thinking about it), they were increasing the likelihood of the depicted events occurring to them.

In the following chapters, we will elaborate on all of these aspects of experience, grounding them in relevant quotes. While the unit of analysis was individual interviews, our analytic focus was on the salient aspects of experience. Here, we are following the phenomenological maxim “back to things themselves” [88]. Thus, these will be presented collapsed across conditions.

We employed a dual task paradigm in order to explore participants’ susceptibility to *demand characteristics*; that is, the particular social dynamic that is established between researchers and participants in the context of a scientific study [109]. This study was advertised as research into emotion regulation. Nevertheless, the patients differed in terms of which aspect of the task was subjectively more important to them. For some, the affective component of the task seemed more salient (a disposition that we coded as *affective focus _L_*_1-_*_L_*_2_):

The emotional task was the more important one yes. It said so in the instructions. […] I perceived it as primary. It took over a larger part of my field of experience. I also found it more challenging.

(EmReg-P-14-T-04)

For an example of *cognitive* *focus _L_*_1-_*_L_*_2_ consider the following:

The cognitive task was the more important. I think because it was more difficult.

(EmReg-P-20-T-03)

The lived experience of the standardized stimuli affected the patients personally (i.e., the emotions had an *egological structure _L_*_1-_*_L_*_2_ (i.e., emotions were about them). Nonetheless, participants reported that they perceived the desired experience to be “horror”, which, in a Slovenian linguistic environment, is an evaluative emotion.

### 3.1. Emotional Response

The prereflective, precognitive experience of the stimuli was characterized by *affective competence _L_*_1-_*_L_*_2_ of the stimulus; i.e., whether the stimuli were capable of eliciting a spontaneous emotional response [96]. For some patients, the static two-dimensional images were *affectively incompetent _L_*_1-_*_L_*_2_:

I did not experience [the stimulus] that intensely. It reminded me of a scene in a movie. It helped me not get too immersed in it. […] It wasn’t as shocking. There is some blood but whatever.

(EmReg-P-14-T-02)

*Affective incompetence _L_*_1-_*_L_*_2_ of the stimuli was commonly associated with the images seeming *artificial _L_*_1_; i.e., they appeared staged, acted out, or too esthetically unpleasing to be capable of eliciting emotions. Furthermore, a handful of patients reported on being aware of having *preregulated_L_*_1_ their emotional response prior to seeing the image even during the emotion engagement condition:

There is a rational blockage. I am able to use it from time to time. I can rationally stop what would otherwise happen on its own. […] It is difficult for me to describe it really. I am simply preventing myself from feeling this ahead of time.

(EmReg-P-26-T-04)

On the other hand, the stimuli could appear as *affectively competent _L_*_1-_*_L_*_2_:

When I saw the slashed up face. The pain is already in the image. It is a human pain. The human life is directly visually connected with me.

(EmReg-P-25-T-02)

*Affectively competent _L_*_1-_*_L_*_2_ stimuli were associated with a *spontaneous emotional response _L_*_2_. This response could be so weak that it did not amount to full and namable emotions (e.g., sadness). Rather, we termed that aspect of experience *deprehension _L_*_1_, a low-level shock experienced during the appearance of the stimulus:

I did not really feel it all that much. So much was going on that it is difficult to parse out what even was the impact of the image. […] It was a very small emotional experience. […] And it went away quickly. […] It was more of a stirring of an emotion. […] Not a lot was going on inside of me.

(EmReg-P-17-T-01)

When the patients experienced *spontaneous emotional response _L_*_2_, the four most common emotions were *anger _L_*_1_, *helplessness _L_*_1_, *sadness _L_*_1_, and *disgust _L_*_1_:

I feel a sense of sorrow. Empathy towards someone. I feel helpless. […] The anger was in my heart. […] Anger rouses me. There is this anxiety to it. Sadness makes me apathetic. I feel helpless.

(EmReg-P-25-T-01)

Occasionally, patients experienced *divergent affect _L_*_1_, an emotional response that was discordant with the nominal valence of the stimuli. Usually, these took the form of curiosity or humor:

There was this boy with a damaged eye. It was pretty interesting. I noticed this detail. There is a tear in his other eye. It was interesting to me how I was able to notice these details.

(EmReg-P-16-T-04)

### 3.2. Constructing an Emotional Response

When the precognitive experience of the stimuli was characterized by *affective incompetence _L_*_1-_*_L_*_2_, the patients felt the need to construct an emotional response. The construction of the emotional response was supported by two phenomenological processes: *empathy _L_*_1-_*_L_*_2_ and *sensemaking _L_*_1-_*_L_*_3_. The experience of *empathy _L_*_1-_*_L_*_2_ consisted of participants attending to the part of their body that was damaged, injured, or diseased in the image. Afterwards, they amplified that embodied experience by imagining how it would have felt like to experience the same type of injury.

On the other hand, we conceptualize *sensemaking _L_*_1-_*_L_*_3_ as an integrated experience consisting of (a) *visual examination of details _L_*_1_; (b) imagining a broader narrative surrounding the image (e.g., what is happening beyond the edges of the image, what were the events leading up to it, and its consequences); and (c) magnification of bodily feelings. The narrative component was a graded experience. Commonly, the starting point was what we termed a *gap in sensemaking _L_*_1_; i.e., being unsure of what the image was depicting. Afterwards, there were different degrees to which participants could make sense of the experience. Here is an example of how *sensemaking _L_*_1-_*_L_*_3_ can be a graded experience:

There is a sense of quote-unquote being filled up by these rational contents and the feeling of bodily connections. There was this linear growth of contents. […] I can feel a gradient of what I can imagine. The hand on the image being my hand. How I would have felt like with a hand like that. It is very clear when I start to look at it where it will go. And with every moment that I stay in that, it is becoming more full and complete. As if I go inside of that feeling […] there is this feeling that will become fulfilled: “It will happen, it will happen. Your heart will start racing, your breath will become less rhythmic. You will feel how it is pushing you inwards.” As if this anticipated state is slowly being fulfilled. And when it is suddenly interrupted, there is this awareness: “Aha, this would have continued on and on and on if I had more time.

(EmReg-P-03-T-01)

For patients, process of *sensemaking _L_*_1-_*_L_*_3_ was *non-linear _L_*_1_. Specifically, as the narrative understanding of the stimulus grows, it is commonly interrupted by doubt, prompting the patients to start the search for the story anew:

I see it and then I have this thought: “Oh, damn. This is basically something else than what you initially thought.” And then it builds up: “You messed it up!” And then, there comes this feeling of guilt. I remember that moment really well. […] It was as if my mind and the screen froze.

(EmReg-P-12-T-02)

When *sensemaking _L_*_1-_*_L_*_3_ was high, we observed three *styles of sensemaking _L_*_2_: *contextual development _L_*_1_, *personal narrative _L_*_1_, and *depressive cosmology _L_*_1_. *Contextual development _L_*_1_ refers to the patients imagining the broader circumstances of the image. This refers to both the events leading up to it and its consequences, as well as how the scene appeared beyond what was depicted in the image. *Personal narrative _L_*_1_ describes situations in which participants tied the stimulus to a memory of something that had happened to them. Finally *depressive cosmology _L_*_1_ is the precognitive experience how the world appears to people and how they feel it works. Without having to think about it, for patients, the world appears as a grim place:

It’s just another bad thing, you know. Another bad thing among the endless bad things that I had seen in my life.

(EmReg-P-26-T-01)

The world and the patients’ way of inhabiting it appear as invariable and incapable of changing:

It really helps me that I don’t have very intense emotions, and if I imagined having them. […] Even when I’m sometimes in phases where I have more […] intense feelings, it wouldn’t work for me to live in this way for longer.

(EmReg-P-17-T-02)

Furthermore, the patients’ self-understanding is characterized by a persistent sense of being different. A patient thus describes *sotto voce* his fondness for animals:

I feel that humans are basically corrupted animals. I feel more sorry for the deer and animals than for people. It’s really hard for me because I see that they are hungry […] Many animals are alone, homeless, living in their own filth, because humans don’t take care of them.

(EmReg-P-13-T-01)

Importantly, while recent developments in enactivism (which claims that cognitive agents do not merely manipulate abstract representations of the outside world in order to guide behavior but enact the world through action–perception coupling [110]) have laid the claim that emotions are actively constituted [111], our participants did not recognize the affective result of such *sensemaking _L_*_1-_*_L_*_3_ as emotions:

We react unconsciously. […] How should I say, non-subjective… So that pure emotion relates to an immediate imprint that the image leaves. Yes, that’s the unconscious.

(EmReg-P-25-T-02)

### 3.3. Disordered Affectivity

The patients reported on two aspects of affective experience that meaningfully contributed to their difficulties with emotion regulation in the context of the cognitive-affective task: *affective enchantment _L_*_1-_*_L_*_2_ and *disintwinement _L_*_1-_*_L_*_2_. These aspects of experience will be explored in detail in the following subsections.

#### 3.3.1. Affective Enchantment

*Affective enchantment _L_*_1-_*_L_*_2_ is an emotional response to the stimuli that is characterized by superstition and high arousal:

I am not looking at the image. The image forces me to look at it. […] It is like the image enchants [Slo. “zacoprati”] you. It keeps you in. […] I don’t do anything. […] It pulls me in. I belong to it. […] I am not doing this consciously. Something simply vibrates.

(EmReg-P-01-T-03)

For a similar example, see the following:

It dragged me away. I felt like it pulled me into this emotional vortex. I started to get dizzy. […] There is this overall feeling of loss of control.

(EmReg-P-16-T-03)

*Affective enchantment _L_*_1-_*_L_*_2_ is associated with the belief that if one looks at an image of a negative event, then something bad will happen:

The most annoying are those images that stay with me. They are potentially real. Bad things that can happen in your life. […] Damn, a car crash. This is worrying. I am actually involved in circumstances where these things can happen. And to it stays with you for a while.

(EmReg-P-18-T-01)

This experience prompted some participants to ignore the instructions in the emotion engagement condition:

I looked at the image entirely without a filter. […] There was a flow of associations that I was creating. And it created a response. […] But then I stopped looking at the image so intensely. I placed a filter on my mind. […] I did not want to immerse myself in the images more than necessary. I am very susceptible to this. To let myself go too much. I know that I have the power to regulate myself. How much of this emotional content can I dose? […] I had been in situations where I was looking at horror movies and I couldn’t finish watching a scene. […] These things tend to follow me for a while.

(EmReg-30-T-01)

One patient described *affective enchantment _L_*_1-_*_L_*_2_ with the Aristotelian term *thaumazein* [An. Gr. “to wonder]:

There is this childish aspect to it all. […] When a child goes out on the street, they are looking at the sky and it starts to rain, they will say: “Wow, it’s raining!” We lose this as adults. We no longer experience wonder at everyday things.

(EmReg-P-29-T-02)

The experience of *affective enchantment _L_*_1-_*_L_*_2_ is long-lasting. Two patients reported in subsequent interviews that the affective experience lasted all day. Thus, a patient on the afternoon following her last interview on the experience of the cognitive-affective task:

I felt a bit anxious. […] For example, when I was washing the dishes, these images […] like those memories. When you remember those images. […] It had something to do with that task. Like something was happening in my brain, like something confused me internally, and I know the system, like how to pull myself together, but it was like I got thrown out of it. And then I had to somehow get myself back together. […] It feels like it was somehow connected to that task itself.

(EmReg-P-10-T-04-02)

As she engaged in this thinking, her reality monitoring became diminished:

I’ve kind of developed these little routines for myself. Like, if I’m holding something in my hand, or have something tangible on my body that I can touch. Because sometimes I’d start having these thoughts where I’d just begin rambling philosophically, like, what if this cup isn’t even real? And then I’d get completely confused. Like, what if I’m already like that, and it’s all just imagined stuff, some kind of hologram.

(EmReg-P-10-T-04-02)

#### 3.3.2. Disintwinement

A polar opposite to *affective enchantment _L_*_1-_*_L_*_2_ is the experience of *disintwinement _L_*_1-_*_L_*_2 _ is a a general experience of being at a distance from the world. A more intuitive name for this would have been *disengagement.* However, “engagement” is used with a wide variety of meanings across the sciences of the mind [112,113,114] and so we opted for a neologism that makes it clear that it does not describe only an attentional or an affective process but an integrated way of being in the world. Consider the following:

I feel separate from the rest of the environment and other people. […] My reactions can be appropriate, but I still feel very far. Like there is water between me and the world. There is an expanse that I feel between here and there. […] [The task] was at a distance. It was so far way that even if it was really intense, it wouldn’t have dragged me with it.

(EmReg-P-08-T-01)

See also the following:

I felt like I was sunk in my head. […] It felt like I was farther away from the world.

(EmReg-P-19-T-03)

The experience of *disintwinement _L_*_1-_*_L_*_2_ is characterized by emotional blunting. However, as already observed by Jaspers (1997) [115], this is not the same as having no emotional response (as in the case of *affective incompetence _L_*_1-_*_L_*_2_ of the stimuli):

There is no feeling of emotions. […] I feel flat. There is nothing. […] It feels like there is empty space in front of me. Something empty. I feel like there is nothing there. […] Where the screen is located, it feels like my whole reality is monochrome. […] I cannot discern what I am feeling, what I am thinking.

(EmReg-P-12-T-01)

In *disintwinement _L_*_1-_*_L_*_2_, patients experience a temporal asynchrony with the cognitive-affective task:

If I compare it to situations where I felt like I was in control, I knew what the next steps are. I knew what I have to do. I trusted in my ability to know how to follow these steps. Whereas now, when I felt like I had lost control, I knew what would follow, but I was not able to really pay attention to the next step. Everything was moving so quickly and in consequence, I did not have as much faith in myself to respond correctly to the task.

(EmReg-P-14-T-03)

For one patient, *disintwinement _L_*_1-_*_L_*_2_ represents a defense against *affective enchantment _L_*_1-_*_L_*_2_:

If I were too open to these things that are around us, you can just cry for 24 h every day. And so, I had to invent strategies for how to become completely numb.

(EmReg-P-05-T-01)

She goes on:

There are these two completely different worlds. In the rational world everything has its “why” and “because.” Everything can be logically explained. Everything can be analyzed. Everything can be put into its place, everything has its own allotted slot. Everything can be ordered using information and intellect. In my emotional world, it is not like that. In a way, things are more chaotic. I have difficulties controlling this world. That is the difference. This world is irrational and it is very intense.

(EmReg-P-05-T-01)

### 3.4. Timing of Emotional Response

Emotional response was characterized by a distinct temporal structure. It varied both in terms of its unfolding in time, as well as the timescales at which it occurred. Despite focusing on the last trial in the interview, the patients reported persistent effects from previous trials as well as events during the day preceding their participation in the study. Three timescales of experience were thus observed: *hour-by-hour _L_*_2_, *minute-by-minute _L_*_2_, and *moment-by-moment _L_*_2_. At the broadest timescale, *affective milieu _L_*_1_ (e.g., mood) was very saliently present for the patients:

My muscles were more tense. I had to invest a lot more energy into being focused. And at the same time, because of that, I feel I didn’t do quite as well on the task. […] I am sitting in a different way. I am kind of unrelaxed. […] My gaze keeps wandering. I had to actively direct it. I needed to keep focusing my mind on one thing.

(EmReg-P-07-T-01)

At the level of *moment-by-moment _L_*_2_ experience, the fixation period was characterized by three mutually exclusive aspects of experience: *anticipation _L_*_1_ (i.e., the preparation for the coming affective stimulus), *persistence _L_*_1_ (where the affective charge from the previous stimulus lingered in their awareness), or *affective baseline _L_*_1_ (the gradual dissipation of the affective charge brought about by the previous stimulus).

For the patients, the affective charge was present already in *anticipation _L_*_1_:

It is the initial phase of crying. When you feel like you are about to start crying.

(EmReg-P-05-T-01)

After an emotional response fully *develops _L_*_1_, it may gradually be *terminated _L_*_1_. Alternatively, it can, as mentioned above, *persist _L_*_1_ in awareness, bleeding into the subsequent trial:

It wasn’t completely gone. […] [During the report period] I can feel it how I felt them then, or at least I can remember how I felt a few seconds ago. […] Sad images never truly went away.

(EmReg-P-15-T-01)

On a *minute-by-minute _L_*_1_ timescale, *persistence _L_*_1_ of the stimuli may be compounded, forming what we termed *impressionability _L_*_1_, the experience of an emotional charge that builds up over the course of performing the affective-cognitive task:

If I am completely honest, I am experiencing the emotions now. So, they come with a delay. […] Before, my experience was very rational. There was this experience of analysis. […] Now, after this delay, I really feel hurt.

(EmReg-P-05-T-01)

### 3.5. Emotion Regulation Strategies

The most commonly reported *emotion regulation strategy _L_*_3_ was *self-command _L_*_1-_*_L_*_2_ wherein patients ordered themselves to regulate their emotions in inner speech. This was followed by *role-taking _L_*_1-_*_L_*_2_; i.e., pretending to be in a professional role within which it makes sense for them to be witnessing such scenes). One account of *role-taking _L_*_1-_*_L_*_2_ provided the title of this paper:

I put myself into this state of *prideful apathy*. Where this will not hurt me. […] The apathy is a sense of hardness. I am creating this hard aura. It is tough, unyielding. It will protect me. There is this initial burst of emotion. I move past that. And then the aura remains solid. I look at the image. There is a corpse there. It does not matter whether it is a real body or a mannequin. No matter what, my response will be apathy. Prideful apathy in which I cannot get hurt.

(EmReg-P-20-T-01)

Further strategies are *breathing and other relaxation techniques _L_*_1-_*_L_*_2_, *swearing _L_*_1-_*_L_*_2_, and *focusing on the cognitive _L_*_1-_*_L_*_2_ (which included the cognitive component of the affective-cognitive task as well as self-imposed tasks such as singing). We further observed *behavioral inhibition _L_*_1-_*_L_*_2_, *distancing _L_*_1-_*_L_*_2_, *reappraisal _L_*_1-_*_L_*_2_, various *ocular strategies _L_*_2_ where the patients modified how they were looking at the image, *structuring one’s time _L_*_1-_*_L_*_2_, and *self-soothing _L_*_1-_*_L_*_2_. Some patients reported on *not having to construct a response _L_*_1-_*_L_*_2_; i.e., when the stimuli were characterized by *affective incompetence*_L1-L2_, participants simply did voluntarily engage in affectivity, thereby precluding the need for regulation. Finally, while *divergent affect _L_*_1_ could be experienced spontaneously, *humor _L_*_1_ was also deployed as an emotion regulation strategy.

Patients commonly remarked that they had learned to engage in explicit emotion regulation strategies either through the process of psychotherapy or by having to deal with difficult feelings for most of their life:

I learned how to do this in psychotherapy. […] I tried to find different ways that would be useful for me. It was a very technical thing for me. Something that would help me get over these feelings.

(EmReg-P-10-T-03)

## 4. Discussion

The goal of the present paper was to describe the lived experience of performing an emotion processing and regulation task in patients with affective disorders. Elsewhere [95], we already put forward a similar phenomenological account in a normative sample, where explicit strategies were rare. The phenomenology of emotion regulation strategies being highly varied and often idiosyncratic is in line with our earlier qualitative observations [95,116]. Furthermore, the patients often reported that the emotion regulation strategies they employ were learned through psychotherapy or years of struggling with affective disorders. This suggests that cognitive processing of emotionally salient stimuli is not different only in degree but kind as well in patients with affective disorders. How therapy restructures one’s lived experience has previously been investigated by Medeiros et al. [117], who demonstrate that a mindfulness-based intervention not only allows people to better regulate stress (specifically ruminative response to stress), but also restructures their overall field of experience.

Our patients experienced the stimuli as more *affectively competent _L_*_1-_*_L_*_2_ (i.e., capable of eliciting a genuine emotional response) [96] than participants from the normative population (whose emotional responses were predominantly experienced as self-constructed) [95]. This observation is in line with widely reported increased sensitivity to negative stimuli in affective disorders, which has been linked to both altered cognitive processes [118,119] and changes in the underlying brain structures [120].

Heightened emotional reactivity was associated with the experience of *affective enchantment _L_*_1-_*_L_*_2_, which is associated with a superstitious belief that if patients are exposed to an image of a negative event, they are more likely to experience such an event in the future. We hypothesize that, *affective enchantment _L_*_1-_*_L_*_2_ is related to *thought–action fusion*, a cognitive bias in which people believe that the mere thought of an action causes its consequences in the real world [121,122,123]. Thought–action fusion is present in all anxiety disorders, especially in states of heightened negative affect [124]. Thought–action fusion is thought to support magical thinking. It has been shown to be associated with increased salience of emotional events [125]. Magical thinking according to Piaget [126] refers to a pre-symbolic organization of cognition. It is characterized by magical substitutions, transanimation of objects and processes, speaking about oneself in the third person, and a collapse between internal and external events. Gregory and Mustata [127] reported on a specific subgroup of adolescents who self-harm (which is a common maladaptive emotion regulation strategy) due to the symbolic value of blood. In a recent network analysis, ref. [128] found that patients with depression and anxiety commonly employ archaic defenses such as splitting (i.e., seeing others as either wholly good or bad) and projective identification (i.e., projecting qualities that are undesirable in oneself onto others). Magical thinking may therefore be of interest as a variable when considering emotion processing in laboratory-based tasks.

*Disintwinement _L_*_1-_*_L_*_2_ (a sense of experiential distance from the stimuli) is closely related to what Fuchs [129,130] refers to as *desynchronization*, a disruption of temporal rhythms (e.g., daily rhythm or menstrual cycle) in depression. *Disintwinement _L_*_1-_*_L_*_2_ further amounts to a general feeling of being detached from the world, for example, a pervasive sense of not being able to engage with shared, intersubjective reality (e.g., at the level of intercorporeality) [131]. Patients with depression often experience a narrowing of possibilities: The world presents itself as a series of obstacles rather than affordances [132]. We can understand *disintwinement _L_*_1-_*_L_*_2_ as disruption of existential feelings [133] in which the emotional meaning of the world is reduced and one’s body feels sluggish and perpetually tired. Desynchronization and disordered intercorporeality are typically investigated in the context of patients’ psychopathological Gestalten; that is, how they experience their disorder as a total alteration of their lifeworld [132]. Our study demonstrates that these alterations are reflected in the performance of cognitive-affective tasks. Thus, in future work, a neurophenomenological link could be established between high-level existential changes and neurocognitive disruptions in affective disorders.

Despite the alteration of basic affective processes described above, some patients did occasionally report on voluntary construction of emotional responses. They often found the stimuli to be unable to elicit genuine emotional responses, citing poor quality of the images themselves as the reason. This critique of standardized libraries of affective images has already been reported in the literature [134]. Patients voluntarily constructed an emotional response through *sensemaking _L_*_1-_*_L_*_3_, an integrated experiential complex consisting of visual examination of details, magnification of bodily feelings, and the construction of a narrative background of the situations depicted in the stimuli. We mapped *sensemaking _L_*_1-_*_L_*_3_ onto appraisal or cognitive evaluation [135,136]. Appraisal involves a person’s assessment of whether or not they can cope with an upcoming emotionally charged situation. Individuals at high risk of developing anxiety tend to judge situations as more threatening than they actually are [137]. Patients in anxious-depressive states tend to chronically underestimate their abilities, leading to feelings of sadness and a defeatist attitude [138], which may have contributed to the *non-linear _L_*_1_ nature of their *sensemaking _L_*_1-_*_L_*_3_.

Our study revealed several methodological considerations for future research in experimental cognitive psychology of emotion dysregulation. Firstly, it may be of interest to explicitly integrate measures of sensemaking into the report procedures of emotion processing tasks. It would be of interest to examine the relationship between subjectively reported arousal and subjectively reported sensemaking. Second, given the preponderance of experience of *affectively competent _L_*_1-_*_L_*_2_ stimuli, it might be of interest to employ works of art that have been explicitly created to elicit strong emotions. Finally, explicit measures of magical thinking in affective disorders could be considered as a covariable to see its moderating effects on subjectively reported arousal. Three qualitative differences were observed in the experience of lived time of patients with affective disorders during the performance of an affective-cognitive task. In anxiety, fear is usually evoked by the uncertainty of the future, whereas in depression, the lived future is characterized by the feeling that a negative outcome is inevitable [139]. While in anxiety, anticipation is associated with avoidant behavior [140], in depression it is associated with the experience of emotions, even if no events are expected [141]. Our participants explicitly reported that *impressionability _L_*_1_ (a gradual, trial-by-trial build-up of emotional charge over each successive trial) was the experience in which rumination took shape [22]. It may be further connected with the phenomenon of *sticky thoughts*, a form of perseverative cognition in which we experience rigid thought patterns that are difficult to disengage [142,143]. In future studies, it would be of interest to see whether trial-by-trial variability in emotion processing and regulation tasks changes depending on participants’ level of ruminating.

### Limitations and Future Directions

As we mentioned above, our goal with the present paper was to put forward a phenomenon-centered account of the lived experience of performing a laboratory-based cognitive-affective task. A quantitative analysis of the relationship between these aspects of experience and specific parameters of the task (e.g., cognitive load and behavioral measures (i.e., arousal and valence)) and their normed values is forthcoming.

The voluntary construction of emotional response reported by our patients opens avenues for future research. Recently, research has been done on *phenomenological control:* the trait-like capacity of people to alter (sometimes unwittingly) their experience in response to verbal suggestions [144,145,146]. Phenomenological control has been shown to modulate several well-known phenomena such as the rubber-hand illusion [147], visually-evoked auditory response [148], mirror-touch synesthesia, and autonomous sensory meridian response [149]. It correlates with subclinical schizotypy [150], which consists of magical thinking [151]. We hypothesize that voluntary construction of affective response during performance of emotion processing will be positively correlated with phenomenological control.

Several limitations of this study warrant addressing. We employed a convenience sample of patients from a single clinic. The clinic is located in the capital city and as such may reflect specific socioeconomic circumstances of urban life. Furthermore, it is possible that some aspects of our patients’ phenomenology are associated with specific cultural factors (e.g., the Slovenian usage of the term “horror”). It would be welcome to see whether researchers from other cultural backgrounds would reach similar conclusions.

In the present study, we observed the lived experience of performing a cognitive-affective task in a cross-sectional manner. It may be that there are differences in the longitudinal dynamics not captured by interviewing patients only at one time point. It would be of interested for future studies to investigate affective phenomenology before and after psychiatric treatment. Relatedly, our findings could be used to develop novel psychological instruments that would allow for the discovery of patterns of emotion dysregulation that cannot be observed with qualitative methods (e.g., isolating phenomenological patterns of dysregulation specific to given diagnostic categories).

Furthermore, studies have shown that while behaviorally there are no differences in the performance of emotion processing tasks before and after treatment with differences in neural dynamics as measured by functional magnetic resonance imagining in schizophrenia [152]. It would be imperative to apply phenomenological methods such as the ones in this paper beyond affective disorders to schizophrenia spectrum disorders to obtain a more comprehensive view of psychopathology of emotions.

## 5. Conclusions

We explored the lived experience of patients with affective disorders engaging in a laboratory-based emotion processing and regulation task. We observed three dimensions according to which the patients’ emotional experience differed: *affective enchantment _L_*_1-_*_L_*_2_, *disintwinement _L_*_1-_*_L_*_2_, and voluntary construction of emotional response, which was mediated by *sensemaking _L_*_1-_*_L_*_3_. Taken together, our findings suggest that laboratory-based cognitive-affective paradigms should be complemented by richer phenomenological assessments (e.g., by accounting for otherwise often ignored aspects of emotional experience, such as vital feelings and feelings of being) [133,153] and possibly redesigned to incorporate more ecologically valid stimuli and task structures that better approximate real-world emotion dynamics and regulation challenges faced by clinical populations. Furthermore, insights from phenomenology may shed light on experiences that are otherwise not associated with a given psychopathology (e.g., magical thinking in depression) but that may confound results in the narrow context of laboratory-based tasks. Finally, the active, constitutive role that people play in their emotions warrants further experimental study. Integrating such phenomenological insights with neuroscientific approaches may lead to more accurate models of emotion dysregulation.

## Data Availability

The annotated codebook including all quotes that were used to construct the categories presented in this paper has been made publicly available at: https://osf.io/qsjua/.

## Supplementary Materials

The annotated codebook and technical details of the stimulus selection can be downloaded at: https://osf.io/qsjua/. References [154,155,156] are cited in the supplementary materials.
